# Supplementary material for: Cell Wall Components and Extensibility Regulate Root Growth in Suaeda salsa and Spinacia oleracea under Salinity
Source: Plants (Basel). 2022 Mar 28;11(7):900. doi: 10.3390/plants11070900 (PMC9002714; doi:10.3390/plants11070900)
Supplement: Supplementary file 1 [file plants-11-00900-s001.zip › plants-1640153-supplementary.pdf]

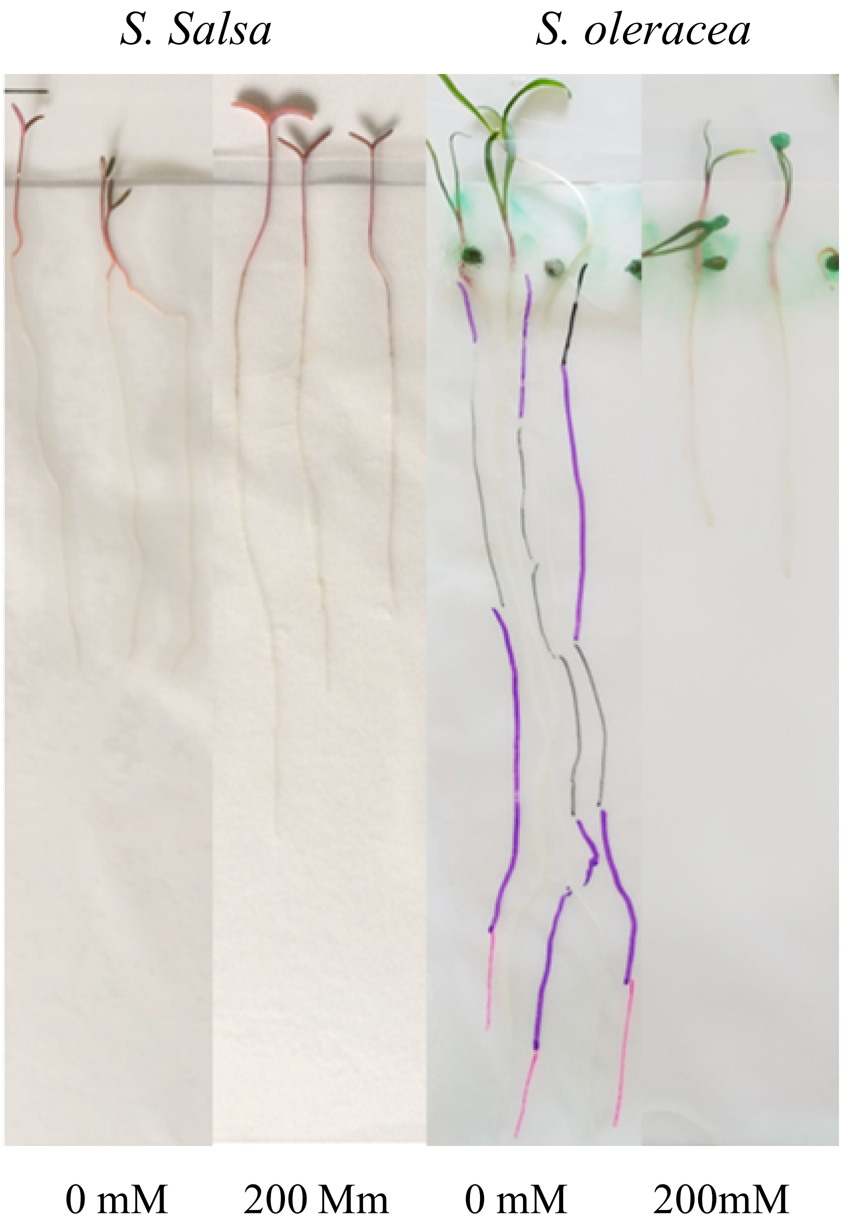

Figure S1. Roots of *Suaeda salsa* and *Spinacia oleracea* 8 days after 0 and 200 mM NaCl treatments.
